# Fat embolism syndrome with patent foramen ovale combined with brain fat embolism and lung fat embolism: A case report

**DOI:** 10.1097/MD.0000000000047255

**Published:** 2026-01-30

**Authors:** Guojun Yang, Jingxuan Xu, Xiaojing Geng, Xudong Qian, Yunpeng Zhang, Fan Sun

**Affiliations:** a Neurosurgery Department, The Affiliated Hospital of Chengde Medical University, Chengde, China; b Department of Neurology, Peking University First Hospital—Miyun Hospital, Beijing, China; c College of Life Sciences and Medicine, Zhejiang Sci-Tech University, Hangzhou, China; d Neurology Department, The Affiliated Hospital of Chengde Medical University, Chengde, China.

**Keywords:** cerebral fat embolism, fat embolism syndrome, patent foramen ovale, pulmonary fat embolism, Three Territory Sign

## Abstract

**Rationale:**

Fat embolism syndrome is a rare complication of fractures or orthopedic surgeries, and cases combined with patent foramen ovale (PFO), cerebral fat embolism, and pulmonary fat embolism are extremely rare.

**Patient concerns:**

A 65-year-old female developed sudden shortness of breath and disturbed consciousness 1 day after hip replacement surgery for a left femoral neck fracture, which was misdiagnosed as an acute cerebral infarction.

**Diagnoses:**

Taking into account the patient’s history of trauma and surgery, cranial diffusion-weighted imaging revealed typical changes indicative of the “3-territory sign”; transthoracic echocardiography confirmed the presence of a PFO; and the transcranial Doppler bubble test detected signals of fat embolism. The patient was ultimately diagnosed with cerebral fat embolism, pulmonary fat embolism, and a PFO.

**Interventions:**

The patient received treatments such as hormone therapy, anticoagulation, and fluid replacement.

**Outcomes:**

Following the treatment, the patient gradually regained consciousness, with respiratory failure corrected and neurological function restored to normal. A follow-up cranial diffusion-weighted imaging scan revealed a marked reduction in the lesions.

**Lessons:**

For high-risk patients with long bone fractures or multiple traumas, close monitoring of vital signs is essential. When shock or neurological changes occur, fat embolism syndrome should be highly suspected. Early fluid resuscitation, timely cranial magnetic resonance imaging examination, and evaluation of pathological shunts (such as PFO) are crucial for improving prognosis.

## 1. Introduction

Fat embolism syndrome (FES) is a rare but potentially life-threatening condition that typically manifests within 24 to 72 hours following fractures or orthopedic surgeries. It is characterized by a triad of respiratory abnormalities (such as dyspnea and hypoxemia), central nervous system disturbances (including altered consciousness, seizures, and focal neurological deficits), and petechial rashes. FES can be categorized into fulminant, complete, and incomplete types, with the latter further divided into pure lung, pure brain, and combined lung-brain presentations. Despite its clinical significance, the pathophysiology of FES remains incompletely understood, involving both physical and biochemical mechanisms. Physical trauma or surgical procedures can cause bone marrow fat and debris to enter the circulation, leading to systemic embolization. Biochemically, the release of inflammatory mediators and free fatty acids contributes to endothelial damage and subsequent organ dysfunction. The presence of a patent foramen ovale (PFO) can complicate the clinical picture by allowing paradoxical embolism, where venous emboli bypass the lungs and directly enter the arterial circulation. This can result in cerebral fat embolism (CFE) and pulmonary fat embolism, further exacerbating the clinical manifestations. Early recognition and prompt management are crucial for improving outcomes in patients with FES.

## 2. Case information

**Figure 1.**
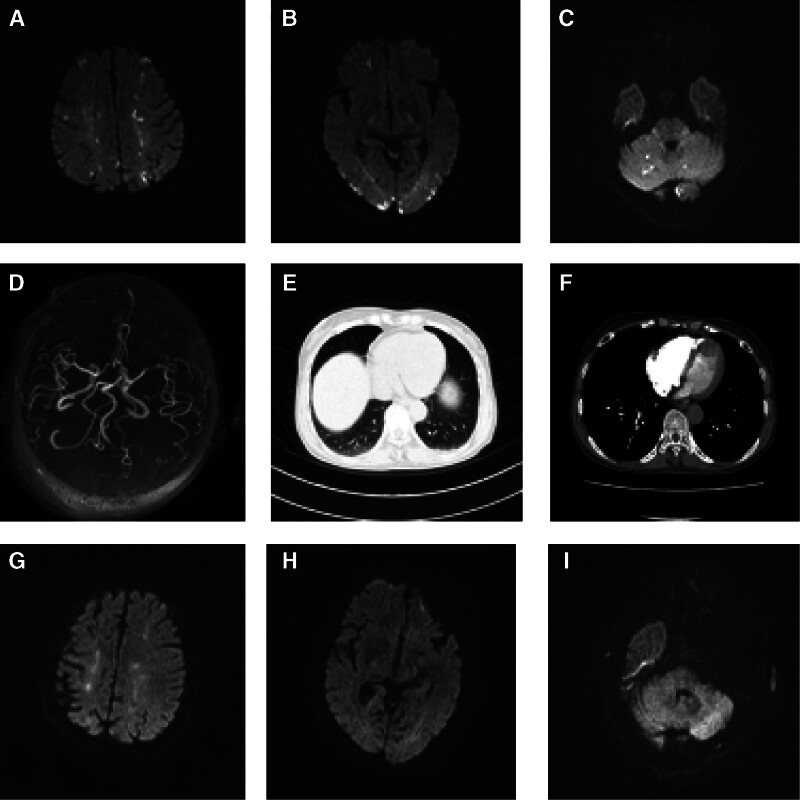
DWI at onset (A–C): Diffuse multiple spot-like hypersignals were observed in bilateral frontal, parietal, temporal, occipital, lobes, center semicovale, and bilateral cerebellum, thereby presenting a typical “Three Territory Sign” change. MRA (D): cerebral artery stiffness and no obvious stenosis. Lung CT (E): Patchy exudate shadows were observed under the pleura in the lower lobes of both lungs with unclear boundaries. Lung CTA (F): Multiple filling defective shadows were observed in some arterial branches of the lower lobe of both lungs. DWI at discharge (G–I): the lesions with limited diffusion were significantly less than before. CTA = computed tomography angiography, DWI = diffusion weighted imaging, MRA = magnetic resonance angiography.

A 65-year-old woman was hospitalized in another hospital for a fracture of the left femoral neck 4 days before admission and underwent hip replacement 3 days after onset. On the first day after surgery, the patient suffered from sudden shortness of breath and disturbance of consciousness. The patient was in a state of lethargy, could open his eyes when called, and showed the following symptoms: slow response, disorientation, slurred speech, and stool incontinence. No limb convulsions were observed, and the symptoms continued without relief. Thus, he was further admitted to the emergency department of our hospital. Head computed tomography (CT) showed multiple cerebral infarction and cerebral white matter degeneration. Diffusion weighted imaging (DWI) of the head showed diffuse multiple spot-like hypersignals in the bilateral frontal, parietal, temporal, occipital lobe, semicovale center, and bilateral cerebellum, thereby presenting a typical “Three Territory Sign” change (Fig. 1A–C). Magnetic resonance angiography showed cerebral artery stiffness, and no obvious stenosis was found (Fig. 1D). Lung CT showed a few patchy exudate shadows under the pleura of the lower lobe of both lungs with unclear boundaries (Fig. 1E). Color doppler ultrasound of both lower limb veins showed no thrombosis. Blood gas analysis (oxygen concentration: 41%, T: 36.5 ℃): pH: 7.45; partial pressure of carbon dioxide: 34 mm Hg; PO_2_: 88 mm Hg; HCO_3_^-^: 23.8 mmol/L; and Lac: 0.7 mmol/L. Oxygenation index was 214, i.e., type I respiratory failure; thus, he was admitted to the neonatal intensive care unit for treatment. On admission, physical examination showed the following: T: 36.3 ℃; P: 92 bpm; R: 19 bmp; blood pressure: 103/52 mm Hg; pulse oxygen saturation: 90%; lethargic state; unclear speech; bilateral pupils perfectly round with a diameter of 2.5 mm; sensitive light reflex; free eye movement; no nystagmus; visible voluntary activities in the limbs; normal muscle tension in the limbs; active tendon reflex in both lower limbs; bilateral pathological signs, negative; meninges stimulation sign, negative; and physical examination of the rest of the nervous system was not cooperative. Audible wet and dry rales were observed in both lungs. Heart rate was 92 bmp (tidiness and no murmur). The abdomen was soft. The liver and spleen were not touched. No tenderness or rebound pain was observed. A slight swelling at the hip joint of the left lower limb was observed, and it was covered by dressing. No edema was found in the right lower limb.

**Figure 2.**
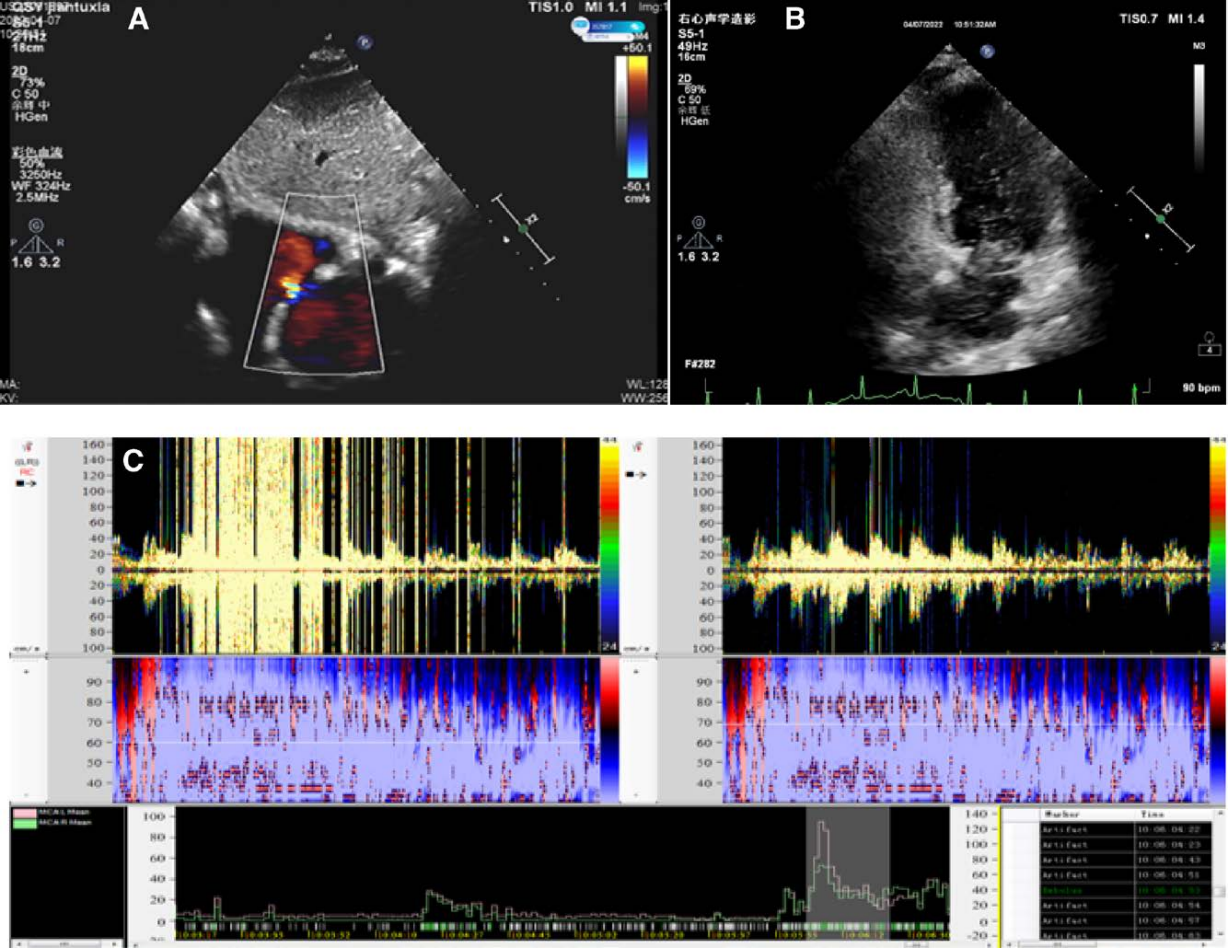
(A–B) TTE showed echo separation at the oval of the atrial septum. The atrial was horizontal and detectable, and left-to-right shunt signal was observed. TTE right echocardiography showed a small amount of contrast agent reflection in the left atrium and left ventricle in the resting state. A large amount of contrast agent reflection was found in the left atrium and left ventricle in the 3 cardiac cycles after Valsalva. TCD foaming test (C): The double-depth blood flow signal of the left vertebral artery was monitored (60–69). The medium embolus signal was detected by pushing the activated saline under the resting state, and rain curtain embolus signal was detected within 12 seconds after Valsalva operation. TCD = trancranial doppler, TTE = transthoracic echocardiogram.

**Figure 3.**
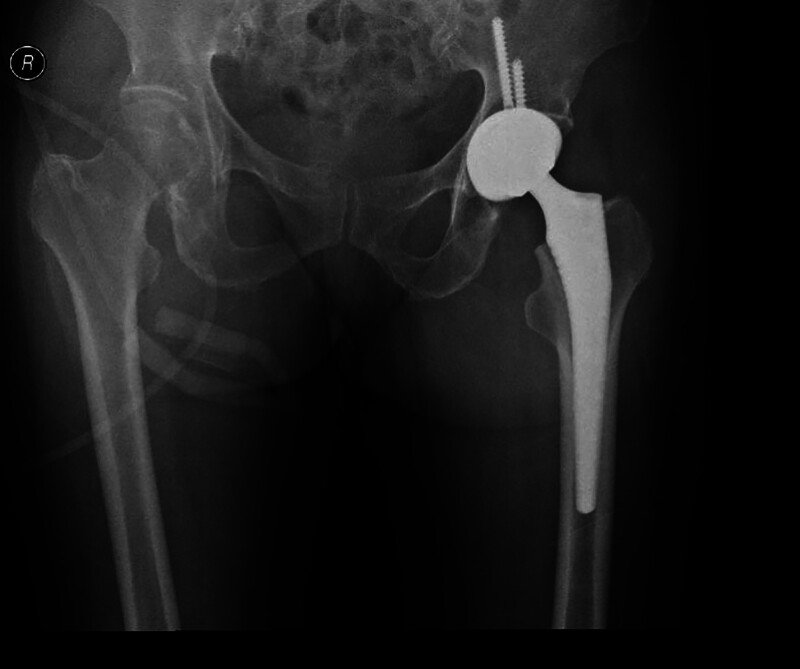
Orthographic hip arthroplasty imaging: After the left total joint replacement, the implanted prosthesis was intact without loosening and displacement, and no bone absorption was observed around it.

Combined with the patient’s history of trauma and the imaging findings of brain and lung, the patient was initially diagnosed as FES and was given drug therapy: methylprednisolone 40mg IVIG qd; human albumin 10g IVIG qd; low-molecular-weight heparin sodium 6000U IH q12h; and normal saline 1500mL IVIG qd. Given that the patient had undergone left femoral neck fracture surgery and cranial magnetic resonance imaging indicated multi-territory infarction, the medical team administered anticoagulant therapy after weighing the risks and benefits. Additionally, high-frequency airway humidization oxygen therapy was provided to correct respiratory failure. After treatment, the patient’s consciousness gradually became clear, and he stopped wheezing. Further improvement of pulmonary computed tomography angiography suggested multiple filling defective shadows in some arterial branches of the lower lobe of both lungs (Fig. 1F). Transthoracic echocardiogram (TTE) showed echo separation in the oval of the atrial septum; atrial was horizontal and detectable, and left-to-right shunt signal was found. TTE right echocardiography showed a small amount of contrast agent reflection in the left atrium and left ventricle in the resting state. A large amount of contrast agent reflection was found in the left atrium and left ventricle in the 3 cardiac cycles after Valsalva (Fig. 2A–B). Trancranial doppler foaming test result was as follows: the double-depth blood flow signal of the left vertebral artery was monitored (60–69), and the medium embolus signal was detected by pushing saline activated under the resting state. After Valsalva, rain curtain embolus signal was detected within 12 seconds (Fig. 2C). The anteropositive radiographs of the hip joint showed that after the left total joint replacement, the implanted prosthesis was intact without loosening and displacement, and no bone absorption was observed around it (Fig. 3). After treatment, the patient’s neurological function returned to normal, and cranial magnetic resonance imaging before discharge showed a significant reduction of diffuse limited lesions (Fig. 1G–I).

## 3. Discussion

The pathophysiological mechanisms of FES are not fully elucidated. The pathogenesis mainly includes physico-mechanical and biochemical aspects. Physical mechanics: after trauma or iatrogenic operation, increased intramedullary pressure forces bone marrow particle, fat, or bone debris into the circulation by open vein.^[1]^ These substances have the potential to promote inflammation and thrombogenesis. When they run by the venous system, they will lead to rapid accumulation of plaque, which will accelerate the formation of fibrin. Then, they eventually will be assembled in the pulmonary circulation. At the same time, the direct toxic reaction of free fatty acids to vascular endothelium leads to the separation of connections between vascular endothelial cells, hemorrhage and edema in the pulmonary stroma, reduction of alveolar surfactant, and impaired lung function.^[2]^ Adipocytes can also enter the arterial circulation through the unclosed foramen ovale or directly through the pulmonary capillary bed, thereby resulting in characteristic nervous system and cutaneous mucosal manifestations. In the trancranial doppler foaming experiment, TTE right echocardiography clearly showed patent foramen ovale, which was the pathological basis of paradoxical embolism. The PFO serves as a direct channel between the venous circulation and the arterial circulation. The blood components of the venous circulation, such as small emboli, enter the arterial circulation through the PFO. The size of PFO, the number of right-to-left shunt, and its structural characteristics are closely related to the occurrence of abnormal embolism. The larger the PFO and the more right-to-left shunts are, the higher the incidence of paradoxical embolism is. The incidence of paradoxical embolism is 2% to 16% of arterial embolism. Transcranial doppler ultrasound can only confirm the presence of an abnormal anatomical structure with right-to-left shunt in the patient, but cannot determine the specific nature of the embolus.

However, a prospective autopsy study showed that pulmonary embolism and other systemic fat embolism are not merely biomechanical events, but biochemical and pathophysiological events.^[3]^ Bronchial and pulmonary arteries have a common network of capillaries in the peribronchial tissue. Such shunt accounts for 5% of all abnormal shunt in a stable internal environment, but this can increase significantly to 15% to 20% in shock or pulmonary fat embolism. Therefore, the authors believe that abnormal shunt plays a more important role than PFO under this particular pathological condition. Biochemical mechanisms suggest that the clinical manifestations of FES can be attributed to an inflammatory reaction. Bone marrow fat is broken down by tissue lipase, resulting in high levels of triglycerides and toxic free fatty acids, thereby causing toxic damage in the pulmonary vascular endothelial cells, which in turn leads to angiogenesis, cytotoxic edema, and bleeding. Damaged pulmonary vascular endothelial cells trigger a cascade of pro-inflammatory cytokines, thereby leading to acute lung injury or acute respiratory distress syndrome. Biochemical studies in patients with FES support this theory with elevated plasma levels of phospholipase A2, pro-inflammatory cytokines (including tumor necrosis factor-α, interleukin-1, and interleukin-6), and free radicals.^[4]^ These pro-inflammatory mediators could explain the mechanism of action of non-traumatic FES, as well as the delay of hours to days from the onset of traumatic factors to the onset of clinical symptoms.

DWI scan of CFE is sensitive and usually shows a “star field pattern” like a diffuse spot-like hypersignals, which is the cytotoxic edema occurring at the early stage of CFE. It has certain specificity for the diagnosis of CFE and is conducive to the early diagnosis and evaluation of the severity of the disease.^[5,6]^ At the same time, DWI can distinguish CFE from other brain diseases, such as trauma, hemorrhage, infarction, hypoxic encephalopathy, diffuse axonal injury, and others. The lesion range of DWI is closely related to the prognosis of patients. However, the patient in the present case presented a typical “Three Territory Sign,” which was symmetrically distributed in bilateral cerebellum, bilateral cerebral ventricles, center of semicovale, and bilateral frontal parietal temporal occipital with multiple patchy diffuse limitations. This may not be related to abnormal shunt–patent foramen ovale. The anatomical evaluation of right-to-left shunt should be improved in patients in which CFE is considered.

At present, specific drugs that directly dissolve the lipid embolus are still lacking. There is no specific treatment for CFE. The main solution is symptomatic supportive treatment, which can do the following: protect important organ functions; correct ischemia, hypoxia, and acidosis; and prevent various complications. Early application of glucocorticoid can reduce the inflammatory response caused by fat emboli, maintain the stability of cell membrane, and reduce cell edema, thus reducing the damage. The patients suffer from pulmonary embolism and were given low molecular heparin anticoagulation treatment. Previous studies suggested that heparin can increase the activity of lipase blood and help remove fat particles in the blood. Another study reports that heparin could enhance the biological activity of the epidermal growth factor, maintain the integrity of the alveolar walls, remove lung edema, and alleviate breathing difficulties. However, activated lipase can increase the concentration of free fatty acids in the blood, and free fatty acids are an important part of the FES pathogenesis. Thus, using heparin to treat FES has potential risks. Free fatty acid often binds with serum albumin in blood, and each gram of albumin can bind 110 mg unsaturated fatty acid to reduce the damage of free fatty acid to lung tissue; this can relieve the respiratory symptoms of patients with FES. Current studies have confirmed that early application of albumin has a therapeutic effect on FES.

## 4. Conclusion

The early clinical symptoms of patients with FES are atypical, and there is no exact laboratory diagnosis criterion. Patients with FES mostly rely on clinical symptoms for early recognition and diagnosis. Therefore, patients at high risk of FES with long bone fracture and multiple trauma should be closely monitored for vital signs. When patients are in shock or show neurological changes, we should be on high alert for FES. Liquid recovery needs to start as soon as possible, to avoid increasing the risk of right to left shunt. And the patients need to achieve complete cranial magnetic resonance in a timely manner. If the imaging changes of “Three Territory Sign” are present, then further investigation is needed to determine whether the patients have pathological anatomical shunt, such as patent foramen ovale and pulmonary arteriovenous fistula.

## Author contributions

**Funding acquisition:** Fan Sun.

**Writing – original draft:** Guojun Yang, Jingxuan Xu, Xiaojing Geng, Xudong Qian, Yunpeng Zhang.

**Writing – review & editing:** Fan Sun.
